# Failure of delayed nonsynaptic neuronal plasticity underlies age-associated long-term associative memory impairment

**DOI:** 10.1186/1471-2202-13-103

**Published:** 2012-08-17

**Authors:** Shawn N Watson, Tara E Risling, Petra M Hermann, Willem C Wildering

**Affiliations:** 1 Department of Biological Sciences, Faculty of Science, University of Calgary, Calgary, AB T2N 1 N4, Canada; 2 Department of Physiology and Pharmacology, Faculty of Medicine, Hotchkiss Brain Institute, University of Calgary, Calgary, AB T2N 4 N1, Canada

**Keywords:** Cognitive impairment, Neural plasticity, Neuronal excitability, Oxidative stress, Lipid peroxidation, α-tocopherol, Mollusc, Classical conditioning, Serotonin, *Lymnaea stagnalis*

## Abstract

**Background:**

Cognitive impairment associated with subtle changes in neuron and neuronal network function rather than widespread neuron death is a feature of the normal aging process in humans and animals. Despite its broad evolutionary conservation, the etiology of this aging process is not well understood. However, recent evidence suggests the existence of a link between oxidative stress in the form of progressive membrane lipid peroxidation, declining neuronal electrical excitability and functional decline of the normal aging brain. The current study applies a combination of behavioural and electrophysiological techniques and pharmacological interventions to explore this hypothesis in a gastropod model (*Lymnaea stagnalis* feeding system) that allows pinpointing the molecular and neurobiological foundations of age-associated long-term memory (LTM) failure at the level of individual identified neurons and synapses.

**Results:**

Classical appetitive reward-conditioning induced robust LTM in mature animals in the first quartile of their lifespan but failed to do so in animals in the last quartile of their lifespan. LTM failure correlated with reduced electrical excitability of two identified serotonergic modulatory interneurons (CGCs) critical in chemosensory integration by the neural network controlling feeding behaviour. Moreover, while behavioural conditioning induced delayed-onset persistent depolarization of the CGCs known to underlie appetitive LTM formation in this model in the younger animals, it failed to do so in LTM-deficient senescent animals. Dietary supplementation of the lipophilic anti-oxidant α-tocopherol reversed the effect of age on CGCs electrophysiological characteristics but failed to restore appetitive LTM function. Treatment with the SSRI fluoxetine reversed both the neurophysiological and behavioural effects of age in *senior* animals.

**Conclusions:**

The results identify the CGCs as cellular loci of age-associated appetitive learning and memory impairment in *Lymnaea* and buttress the hypothesis that lipid peroxidation-dependent depression of intrinsic excitability is a hallmark of normal neuronal aging. The data implicate both lipid peroxidation-dependent non-synaptic as well as apparently lipid peroxidation-independent synaptic mechanisms in the age-dependent decline in behavioural plasticity in this model system.

## Background

Age-associated learning and memory impairment is a signature of the normal aging process in humans and animals of diverse phylogenetic background [1-6]. In contrast to age-associated neurodegenerative diseases, functional decline due to normal brain aging involves subtle physiological changes in neuron and neuronal network function rather than a widespread loss of neurons [6-10]. Understanding these processes is essential to develop effective strategies to deal with the problems associated with old age. Yet, molecular and cellular dimensions of the normal brain aging are still incompletely understood. In the current study we continue our exploration of the hypothesis that declining performance of the aging brain is associated with a reduction in neuronal electrical excitability resulting from oxidative stress occurring at the level of the neuronal plasmamembrane. The neuronal plasmamembrane, due to its high poly-unsaturated fatty acid (PUFA) content, is particularly prone to non-enzymatic oxidative insult [11-13]. Moreover, the idea that oxidative stress is an important agent of biological aging of the brain has substantial support [14-18] and a growing body of evidence suggests that a progressive decline in intrinsic electrical excitability of aging neurons is a significant aspect of the problem [3,6-10].

In our studies we use the pond snail *Lymnaea stagnalis* as model system of age-associated memory impairment [6,19]. Gastropod mollusks like *Lymnaea* have a relatively simple nervous system containing many identified neurons and precisely mapped, functionally characterized neuronal circuits that allow one to trace the neural basis of whole animal behavioural and physiological functions to the level of single neurons and synapses [19]. In the current study we utilize an established and widely studied classical appetitive reward-conditioning paradigm involving chemosensory conditioning of the animals’ feeding behaviour (i.e., “rasping”) to investigate neurophysiological correlates of age-associated changes in learning and memory abilities [6,20-24]. The neurobiological substrate of *Lymnaea’s* feeding behaviour and important details of the mechanisms of plasticity underlying the expression of appetitive long-term memory (LTM) are known in detail [24,25]. Specifically, the expression of associative LTM depends on presynaptic facilitation arising from a behavioral conditioning-induced persistent depolarization of a pair of state-controlling serotonergic interneurons, the cerebral giant cells (CGCs) that play a permissive role in feeding behavior but have no direct role in the generation of the rhythmic feeding pattern [24]. The persistent depolarization affects the synaptic outputs of the CGCs within the cerebral ganglia. This underlies the presynaptic facilitation of the sensory pathways to the feeding command interneurons. One of its consequences is that throughput of peripheral chemosensory afferent information to the feeding circuit is altered [24].

Our previous work has linked age-associated appetitive LTM impairment in *Lymnaea stagnalis* to a reduction in intrinsic excitability of the CGCs [6]. This change in CGC response characteristics is of a magnitude likely to upset the cells’ control functions and, possibly, their ability to express the behavioral conditioning-induced persistent depolarization underlying appetitive LTM in this model system [6]. Recent studies have linked age-associated decline of electrical excitability of another identified *Lymnaea* neuron to lipid peroxidation [3,19]. These studies also indicate that this neuro-physiological effect of age is reversible by treatment with the lipophilic anti-oxidant α-tocopherol (vitamin E) [3,19]. Alpha-tocopherol is the main anti-oxidant agent operating in the lipid bilayer domain and a cell’s primary defense against the progression of lipid-peroxidation. The current study examines whether the finding that treatment with α-tocopherol reverses electrophysiological phenomena associated with old age can be extrapolated to the CGCs and whether such correction is sufficient to restore appetitive learning and memory capability of old *Lymnaea* to the level typical of young animals.

To this end we compared learning abilities and CGC electrophysiological parameters of old LTM-impaired animals that were put on a α-tocopherol enriched diet with similar animals that were fed a standard diet with a lower α-tocopherol content. In addition, mindful of the notion that LTM deficiency in this model possibly arises from a failure of serotonin release by the CGCs, we tested the impact of the selective serotonin reuptake inhibitor (SSRI) fluoxetine on the same electrophysiological and behavioural parameters in old LTM-impaired animals. The data we present here reveals an intriguing difference in α-tocopherol’s and fluoxetine’s ability to reverse electrophysiological and behavioural effects of age. Our data suggests that restoration of neuronal electrophysiological response characteristics to a young mature condition alone is not sufficient to return appetitive behavioural plasticity of senescent animals to the receptive state of younger animals. As discussed, the data provides arguments that lipid peroxidation-dependent non-synaptic as well as lipid peroxidation-independent synaptic factors play a role in this model system of age-associated learning and memory impairment.

## Results

### Age-associated appetitive LTM breakdown correlates with failure of behavioural-conditioning induced CGC depolarization

**Figure 1:**
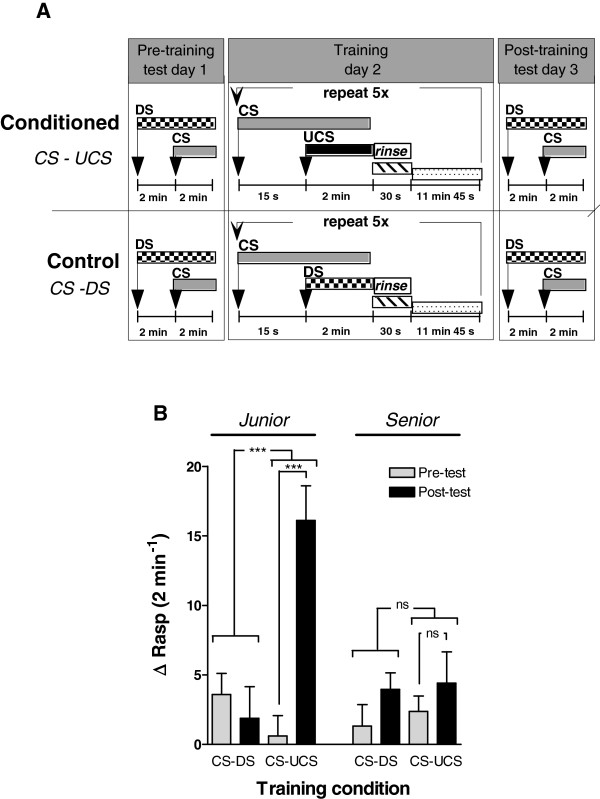
**Long-term memory assessment in *Junior *and *Senior *animals. A: ** Long-term memory performance was determined using a single day, multi-trial, non-aversive, appetitive conditioning assay. On day 1, prior to behavioural conditioning animals were tested (“Pre-training test”) by the addition of a disturbance stimulus (DS, pond water), counting the number of rasps for 2 minutes followed by the application of the conditioned stimulus (CS, 4 ppm n-amyl acetate) and counting the number of rasps over the following 2 minutes. Conditioning occurred on day 2 and consisted of pairing the CS with the unconditioned stimulus (UCS; 0.4% sucrose) which was repeated 5 times. A control (unconditioned) group received a similar timed combination of CS and DS. On day 3 animals were subjected to a post-training test protocol identical to the pre-training test conditions. **B:***Junior* and *senior* animals were randomly assigned to either control or conditioned groups. There was a robust response to conditioning (CS-UCS) in *junior* animals as displayed by a significant increase in the Δrasp values in the post-training test. In contrast, none of the conditioned *senior* animals and unconditioned (CS-DS) control *junior* or *senior* animals responded with significant feeding movements in the post-training test. These results are indicative of LTM impairment in *senior* snails. *** = p < 0.001, ns = non-significant.

Our central hypothesis holds that age-associated appetitive LTM failure involves a decline in electrical excitability of the CGCs resulting in a loss of these cells’ capacity for learning induced persistent membrane depolarization. To assess this idea, LTM of *junior* and *senior* snails was evaluated using a single-day, multi-trial, non-aversive appetitive-conditioning assay (Figure1A) followed by electrophysiological evaluation of CGC excitability in the isolated CNS.

*Junior* (n = 35) and *senior* (n = 43) snails were assigned at random to two test groups. One group was submitted to behavioural conditioning by means of paired application of amyl acetate (the conditioned stimulus or CS) and sucrose (the unconditioned stimulus or UCS). Animals treated this way will be denoted by the abbreviation CS + UCS throughout the text. The second group functioned as control for stimulus application. In this case the UCS stimulus was replaced by application of water (i.e., vehicle-only disturbance control or DS). Animals treated in this manner are signified by the abbreviation CS + DS.

As observed in our previous study [6], there was a significant interaction between age and behavioural conditioning (Figure1B; Age x Training x Δrasp, F_1,74_ = 13.779, p < 0.001). This interaction arose from the fact that *junior*, but not *senior* animals displayed robust conditioned feeding responses after conditioning (CS + UCS) compared to their aged-matched controls (F_1,74_ = 23.275, p < 0.0001 and F_1,74_ = 0.0347, p = 0.85 for *junior* and *senior* animals respectively).

**Figure 2:**
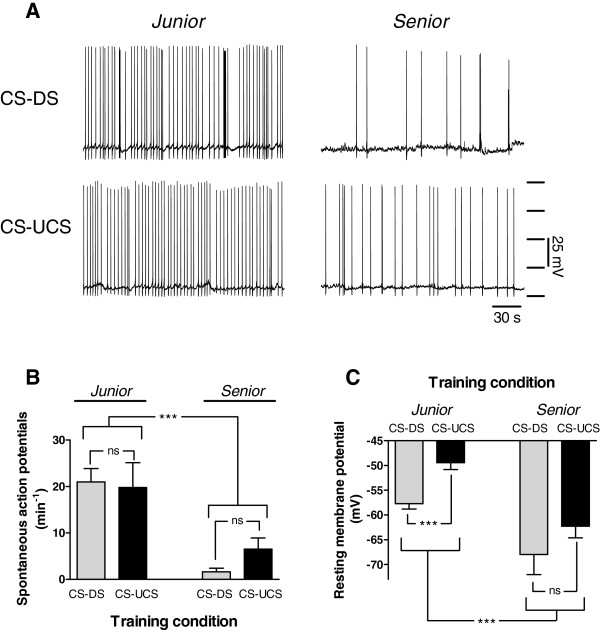
**Electrophysiological assessment of CGCs in *Junior *and *Senior *animals. A. ** Examples of intracellular recording of spontaneous electrical activity in CGCs of *junior* and *senior* conditioned (CS-UCS) and unconditioned control (CS-DS) animals. **B.** Average number of spontaneous action potentials of CGCs in both *junior* and *senior* conditioned and control animals. Aged CGCs showed a significantly lower spontaneous action potential firing rate than *junior* CGCs No significant difference was observed between the control and conditioned groups within each age category. **C.** Resting membrane potential of *junior* CGCs was more depolarized compared with *senior* CGCs. Behavioural conditioning further depolarized resting membrane potential in *junior* but not in *senior* animals. * = p < 0.05, *** = p < 0.001, ns = non-significant.

Electrophysiological assessment of the CGCs in these animals after training revealed substantial differences in resting membrane potential and spontaneous activity between test groups (Figures2A, B and C). Spontaneous action potential activity differed significantly between *junior* and *senior* animals but not between the behaviourally conditioned animals and their aged-matched controls (Figure2B; Age x Training F_1,41_ = 25.526, p < 0.001; and Training within age F_1,41_ = 0.068, p = 0.80 for *junior* CNS’s and F_1,41_ = 1.179, p = 0.28 for *senior* CNS’s respectively).

On average, the resting membrane potential of CGCs was more hyperpolarized in s*enior* CNS’s than that of their *junior* counterparts independent of their behavioural training status (Figure2C; n = 45, Mann–Whitney Z = 3.595, p < 0.001). Moreover, comparison of resting membrane potentials measured in CGCs of CNS preparations from behaviourally conditioned (CS + UCS) with their respective control groups (CS + DS) within each of the age groups, showed that CGC resting membrane potential of conditioned (CS + UCS) *junior* snails was more depolarized compared to the *junior* control (CS + DS) animals (Figure2C; n = 23, Mann–Whitney Z = −3.728, p < 0.001). In contrast, behavioural conditioning had no effect on CGC resting membrane potential in *senior* snails (Figure2C; n = 23, Mann–Whitney Z = −0.894, p = 0.37). Together, these data are consistent with the ideas proposed by Kemenes et al. [24] that training-induced depolarization of the CGC is an element of LTM in this appetitive learning model and point towards breakdown of this mechanism as a factor in appetitive LTM impairment observed in aged snails.

### Dietary supplementation of α-tocopherol reverse effect of age on CGC excitability but fails to rescue appetitive LTM

**Figure 3:**
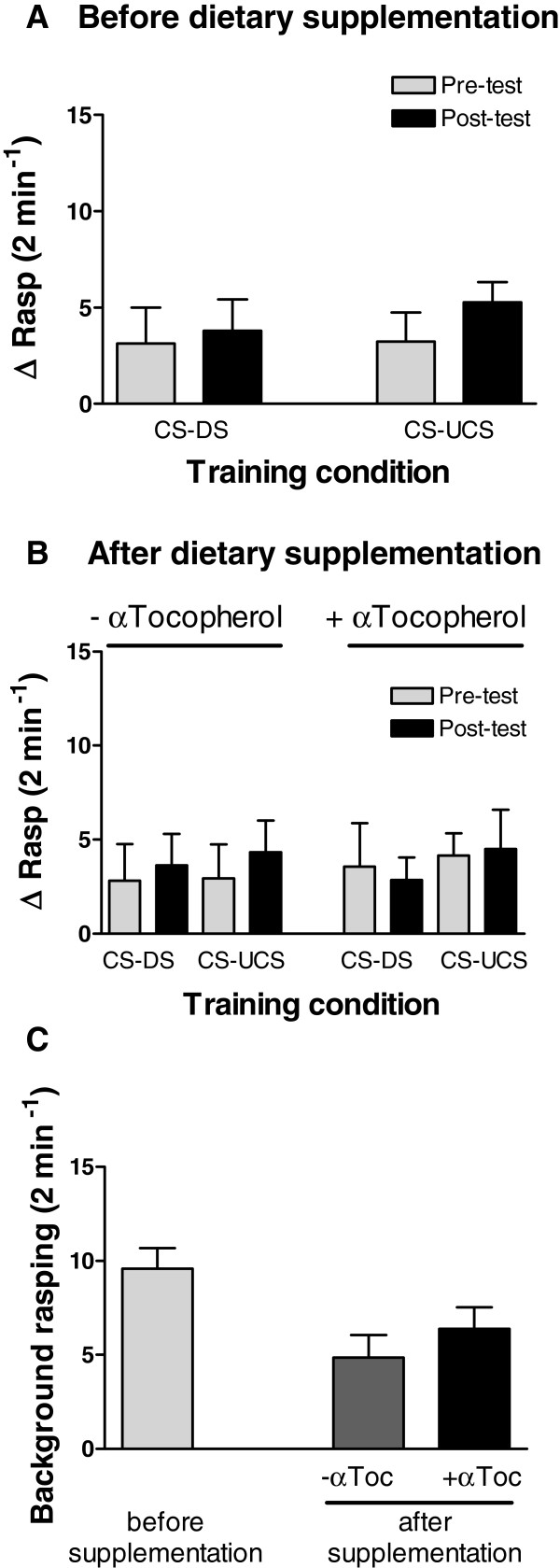
**Effect of dietary α-tocopherol supplementation on LTM performance in Effect of dietary α-tocopherol supplementation on LTM performance in *senior *animals. A **. Long-term memory assessment of *senior* snails before the start of dietary supplementation with α-tocopherol. None of the control (CS-DS) or conditioned (CS-UCS) animals displayed a significant increase in the ΔRasp value indicative of LTM impairment. **B.** Long-term memory assessment of *senior* snails following 2 months of α-tocopherol dietary supplementation (+ αTocopherol) or vehicle only (− αTocopherol). Neither α-tocopherol or vehicle treated animals displayed an improvement in LTM performance **C.** Comparison of spontaneous background rasping rates reveals no significant differences between animals treated with α-tocopherol (+ αToc) or vehicle only (−αToc).

The data presented above show that appetitive LTM failure in aged animals is associated with a very prominent reduction in action potential activity in the CGC’s. Previous studies suggest that the suppression of action potential activity observed in aged *Lymnaea* neurons is associated with a trend towards more prominent action potential accommodation linked to progressive lipid peroxidation [3]. As a consequence neurons can no longer support sustained periods of elevated spiking activity. Thus, stimulus response characteristics of *Lymnaea* neurons likely change with age, possibly to the extent that mechanisms of activity-dependent plasticity are affected. Since treatment of aged isolated CNS’s with α-tocopherol overturned the age-associated drop in neuronal excitability we tested this compound’s efficacy in restoring appetitive LTM function of old animals. For this purpose, 17 aged animals were fed a α-tocopherol enriched diet for two months while another group of 17 animals was fed the same diet without additional α-tocopherol. Behavioural assessment confirmed that all animals were LTM deficient prior to dietary intervention (Figure3A; F_1,32_ = 0.199, p = 0.66). Spontaneous background rasping frequency declined slightly but significantly over the course of the two month dietary intervention due to an, at this moment unknown cause. Importantly, this effect was independent of the dietary composition the animals were fed (Figure3C; Diet x Time F_1,32_ = 0.043, p = 0.84; Time F_1,32_ = 9.950, p < 0.001; Diet F_1,32_ = 0.736, p = 0.40). Contrary to our expectations, dietary supplementation of α-tocopherol failed to restore LTM function in these aged animals (Figure3B; Treatment x Training F_2,28_ = 0.018, p = 0.98). 

**Figure 4:**
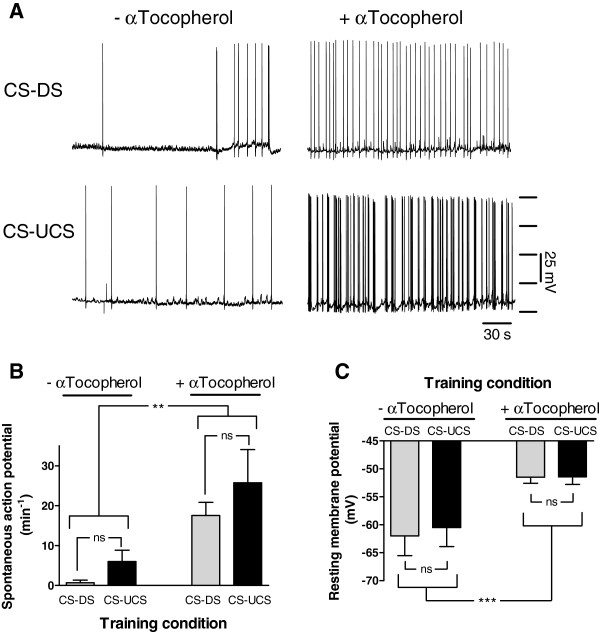
**Effect of α-tocopherol treatment on electrical activity CGCs. A.** Examples of intracellular recording of spontaneous electrical activity in CGCs of control (CS-DS) and conditioned (CS-UCS) *senior* animals with supplementation of vehicle only (− αTocopherol) or with α-tocopherol (+ αTocopherol). **B.** Average number of spontaneous action potentials of CGCs in vehicle only and α-tocopherol treated conditioned and control animals. α-tocopherol caused an significant increase in the number of spontaneous action potentials recorded from CGCs compared with CGCs taken from vehicle treated animals. No significant effect was observed of conditioning on spontaneous electrical activity in both dietary treatments. **C.** CGC resting membrane potential of α-tocopherol treated snails was significantly more depolarized compared with vehicle only treated animals. Behavioural conditioning had no effect on resting membrane potential in both treatments. * = p < 0.05, ** = p < 0.01, *** = p < 0.001, ns = non-significant.

Yet, intracellular recordings performed on isolated CNS’s dissected from these same animals after completion of the behavioural trials revealed a positive effect of diet on action potential activity of CGC’s (Figures4A and B, Diet, F_1,20_ = 9.511, p < 0.01) and CGC resting membrane potential (Figure4C; Mann–Whitney Z = −3.430, p < 0.001). No evidence was found of an effect of behavioural conditioning on either CGC spiking activity (Figure4B; F_1,20_ = 1.297, p = 0.27) or resting membrane potential (Figure4C; Mann–Whitney; Z = −0.614, p = 0.54 and Z = 0.508, p = 0.61 for vehicle-only and α-tocopherol treated animals respectively). Thus, feeding aged animals a α-tocopherol enriched diet appears to restore excitability and resting membrane potential of old CGCs to a condition found in young LTM-competent animals, but failed to rejuvenate appetitive LTM functionality.

Aged animals do normally not show any motor and/or chemosensory deficits [6]. However, it is possible that dietary supplementation with α-tocopherol affects chemosensory and/or motor functions. To test this possibility a separate set of aged animals were fed an α-tocopherol enriched diet for two months while a control group was fed the same diet without additional α-tocopherol. Subsequently, we determined the feeding response upon sucrose application and measured by means of extracellular recording techniques the activity of afferents in the superior lip nerve in a semi-intact preparation before and after amyl acetate application.

**Figure 5:**
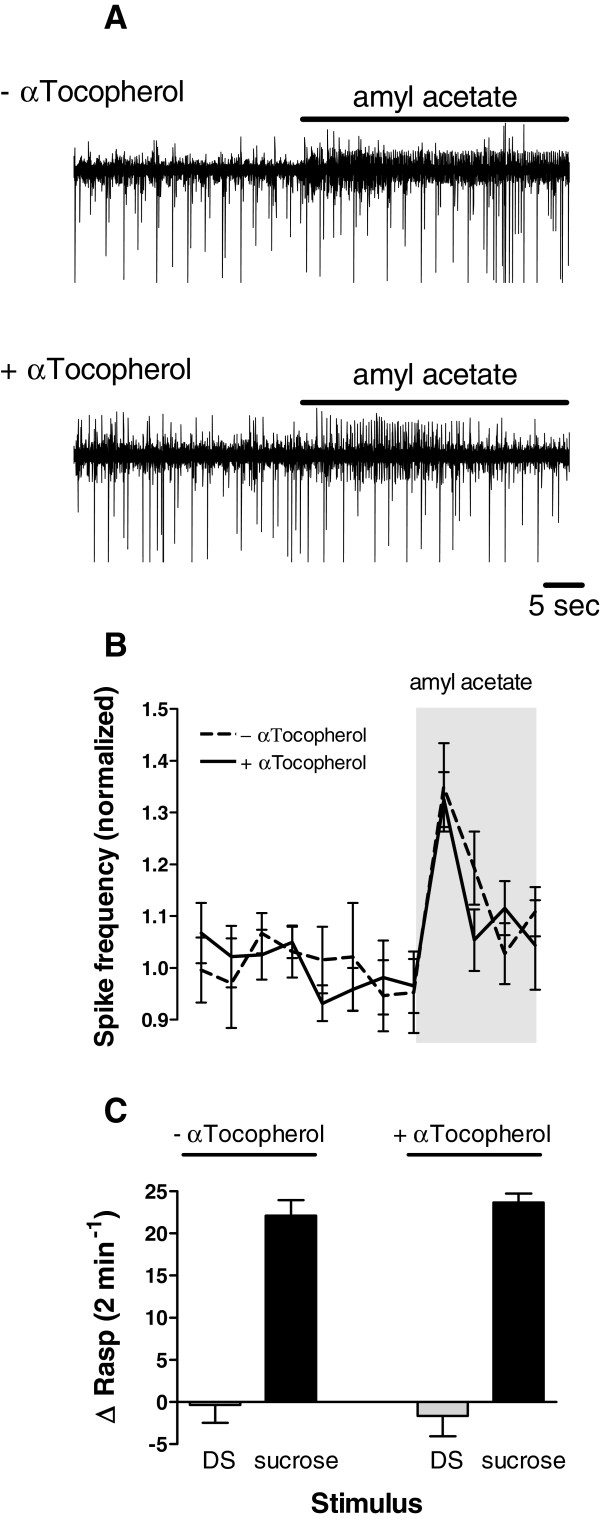
**Effect of dietary α-tocopherol supplementation on chemosensory and motor function in *senior *animals. A. ** Examples of extracellular recording of afferents in the superior lip nerve before and after application of n-amyl acetate (4 ppm) of *senior* animals with dietary supplementation of vehicle only (− αTocopherol; top trace) or α-tocopherol (+ αTocopherol; bottom trace). **B.** Average action potential frequency (bin size 30 sec) of afferents in superior lip nerve 4 minutes before and 2 minutes during amyl acetate application. There is no difference in response upon n-amyl acetate application between the two different treatment groups **C.** Average change in the number of rasps evoked by application of pond water (DS) or sucrose (0.4%). Only sucrose induced a robust but similar response in *senior* animals fed with dietary supplementation of vehicle only (− αTocopherol) or α-tocopherol (+ αTocopherol).

Figure5 shows that dietary supplementation with α-tocopherol did not affect sensory perception of amyl acetate. Application of amyl acetate (4 ppm) induced an immediate and significant increase in electrical activity of the afferents in the superior lip nerve. This effect was similar in both treatment groups (n = 8 for both groups, Figures 5A, B; ANOVA time vs treatment F_1,14_ = 0.841, p = 0.37; contrast - α-tocopherol pre application vs post application F_1,14_ = 18.489, p < 0.001; contrast + α-tocopherol pre application vs post application F_1,14_ = 9.017, p < 0.01). In addition, examining rasping responses after sucrose stimulation in intact animals showed that diet had no effect on chemosensory or motor function. That is, while none of the animals responded with rasping movements after application of the disturbance stimulus (DS), application of sucrose (0.4% final concentration) induced a robust feeding response in both α-tocopherol fed animals (n = 9) and their controls (n = 9; Figure5C; ANOVA solution vs treatment F_1,16_ = 0.412, p = 0.53; contrast sucrose response α-tocopherol vs control F_1,16_ = 0.539, p = 0.47). Thus, dietary supplementation with α-tocopherol does not affect feeding network related chemosensory or motor functions.

### Fluoxetine treatment reverses effects of age on appetitive LTM and CGC excitability

The data presented in the preceding sections are consistent with conclusions of Kemenes et al. [24] that CGC depolarization is both a necessary and sufficient condition for the expression of appetitive LTM in non-senescent animals [24]. However, the above observation that dietary intervention succeeded in recreating the electrophysiological conditions in old animals normally associated with appetitive LTM, yet did not restore LTM functions suggest a failure of additional mechanisms involved in depolarization-induced presynaptic facilitation of CGC terminals in old animals. This idea is consistent with evidence presented by Patel et al. that CGC excitation-secretion coupling characteristics change with age [26]. Prompted by the latter authors evidence that the SSRI fluoxetine appears to reverse this effect of age, we tested this compound’s ability to reverse the effects of age on appetitive LTM function and CGC electro-physiological characteristics of old LTM-impaired animals.

In these experiments 30 *senior* animals received before behavioural conditioning a single 50 μl injection of fluoxetine in their body cavity to create an effective hemolymph concentration of fluoxetine of ~100nM. A second group of 30 *senior* animals subjected to the same experimental protocol received vehicle-only injections (i.e., water). Behavioural and electrophysiological assessments of these animals proceeded as described previously.

**Figure 6:**
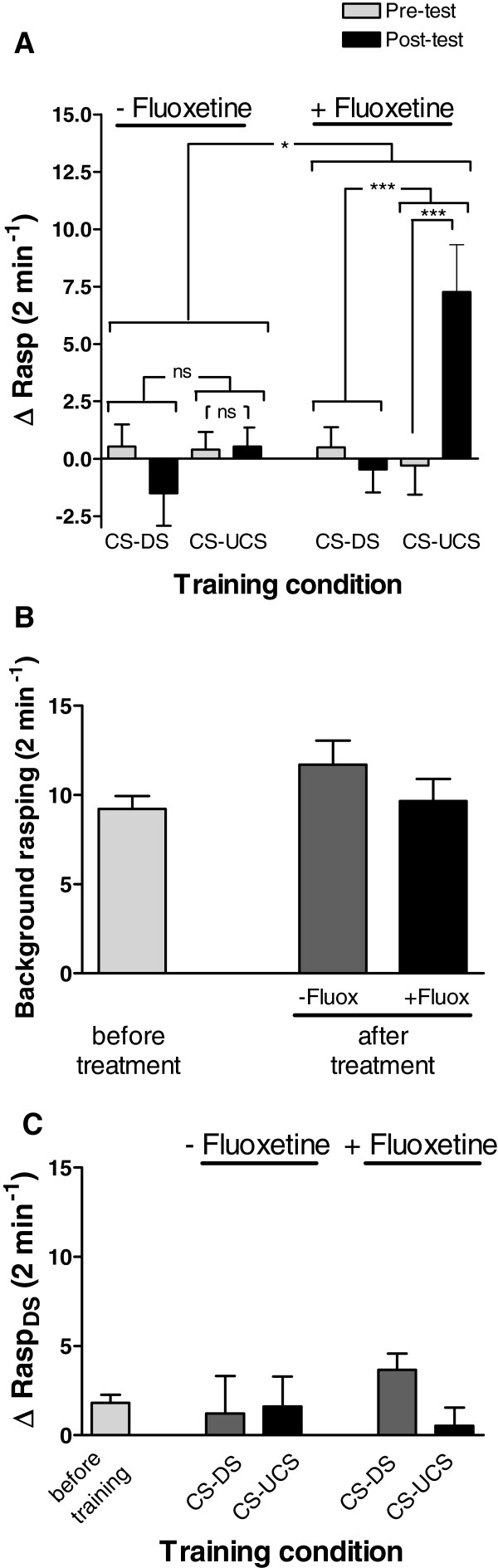
**Effect of fluoxetine treatment on LTM performance in *senior *animals. A.** Long-term memory assessment of *senior* snails following treatment with fluoxetine (+ Fluoxetine) or vehicle only (− Fluoxetine). Fluoxetine treatment significantly improved LTM performance in conditioned (CS-UCS) *senior* snails. In contrast, vehicle only treated conditioned animals showed no indication of LTM. **B.** Comparison of spontaneous background rasping rates reveals no significant differences between animals treated with fluoxetine (+ Fluox) or vehicle only (−Fluox). **C.** Comparison of rasping response after application of the disturbance stimulus reveals no significant differences between unconditioned (CS-DS) and conditioned (CS-UCS) animals regardless whether they were treated with fluoxetine (+ Fluox) or vehicle only (−Fluox). * = p < 0.05, *** = p < 0.001, ns = non-significant.

Fluoxetine-treated behaviourally-conditioned (CS-UCS) animals displayed robust conditioned feeding responses, while no such response was observed in the vehicle-only injected conditioned animals or all unconditioned (CS-DS) animals (Figure6A; Treatment x Training x Δrasp, F_1,55_ = 4.371, p < 0.05; Training x Δrasp within fluoxetine treated animals, F_1,55_ = 15.997, p < 0.001; Δrasp pre-training vs Δrasp post-training for fluoxetine treated CS-UCS group, F_1,55_ = 25.157, p < 0.0001; Training x Δrasp within vehicle- treated animals, F_1,55_ = 0.998, p = 0.32; Δrasp pre-training vs Δrasp post-training for vehicle-treated CS-UCS group, F_1,55_ = 0.008, p = 0.93). Importantly, fluoxetine injection had no effect on spontaneous baseline rasping frequency (Figure6B; Treatment x Time F_1,57_ = 0.033, p = 0.86; Time F_1,57_ = 3.334, p = 0.07; Treatment F_1,57_ = 1.6805, p = 0.20). However, it is still remotely possible that Fluoxetine-treated behaviourally-conditioned (CS-UCS) animals increased their feeding movements due to non-associative effects. If this is the case, one would expect after training an increase in the number of rasps due to for instance handling, changing environment and/or upon application of a (neutral) solution. Yet, comparing the feeding response upon DS application (i.e., ΔRasp_DS_ = #rasps after DS - #rasps before DS) before and after training indicate no significant changes in the number of rasps in the unconditioned (CS-DS) or the conditioned (CS-UCS) animals in either of the two treatment groups (Figure6C; Time x Treatment x Training F_1,55_ = 1.876, p = 0.18; Time x Treatment F_1,55_ = 0.736, p = 0.39; Time x Training F_1,55_ = 0.041, p = 0.84). Together, this clearly indicates that handling, a change in environment nor the application of a solution (DS) induces non-associative feeding responses. Thus, only in (Fluoxetine) conditioned animals (CS-UCS) and only application of the conditioned stimulus is capable of evoking a significant increase in rasping movements.

**Figure 7:**
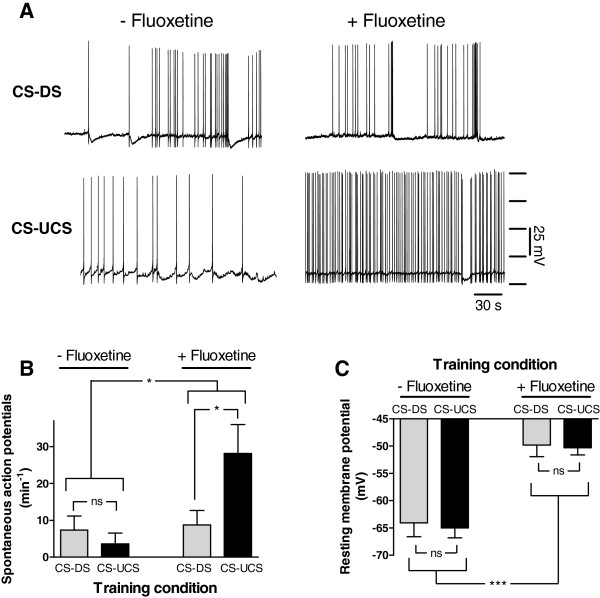
**Effect of fluoxetine treatment on electrical activity CGCs. A.** Examples of intracellular recording of spontaneous electrical activity in CGCs of control (CS-DS) and conditioned (CS-UCS) *senior* animals treated with vehicle only (−Fluoxetine) or with fluoxetine (+ Fluoxetine). **B.** Average number of spontaneous action potentials of CGCs in vehicle only and fluoxetine treated conditioned and control animals. Fluoxetine caused a significant increase in the number of spontaneous action potentials but only in CGCs from conditioned (CS-UCS) animals. No significant effect on electrical activity was observed in fluoxetine treated unconditioned control animals (CS-DS) or vehicle treated conditioned and unconditioned animals. **C.** CGC resting membrane potential of fluoxetine treated snails was significantly more depolarized compared with vehicle only treated animals. Behavioural conditioning had no effect on resting membrane potential in both treatments. * = p < 0.05, *** = p < 0.001, ns = non-significant.

Electrophysiological assessment of CGC excitability proceeded after completion of the behavioural evaluation (i.e., >24 hrs after fluoxetine injection and training). These tests revealed that injection of fluoxetine caused a selective increase in spontaneous electrical activity in behaviourally conditioned animals (Figure7). That is, mean CGC spiking activity was significantly raised above the levels in the other test groups in the fluoxetine-treated behaviourally conditioned animals only (i.e., CS-UCS; Figure7B; Treatment x Training, F_1,36_ = 4.797, p < 0.05; CS-DS vs. CS-UCS within vehicle treated animals, F_1,36_ = 0.243, p = 0.63; CS-DS vs. CS-UCS within fluoxetine-treated animals, F_1,36_ = 4.797, p < 0.05). In addition, fluoxetine induced a marked depolarization of CGCs in both behaviourally conditioned and disturbance control animals that was not observed in their vehicle-treated counterparts (Figure7C; Mann–Whitney, Treatment x Training, Z = −4.826, p < 0.0001; CS-DS vs. CS-UCS within vehicle treated animals, Z = − 0.359, p = 0.72; CS-DS vs. CS-UCS within fluoxetine-treated animals, Z = 0.420, p = 0.67).

## Discussion

In the current study we show; 1- A single session, five trial classical appetitive reward-conditioning procedure induced robust associative LTM in mature animals in the first quartile of their lifespan but failed to induce such memory in animals in the last quartile of their lifespan; 2- Appetitive LTM formation correlated with resting membrane potential depolarization of modulatory interneurons (CGCs) with a critical function in the expression of appetitive LTM; 3- CGCs of *senior* animals fired significantly fewer action potentials and were significantly hyperpolarized relative to their younger counterparts in CNSs dissected from both behaviourally conditioned and control animals; 4- Dietary supplementation of *senior* animals with α-tocopherol reversed the effect of age on action potential activity and resting membrane potential of CGCs in a behavioural conditioning-independent manner yet failed to restore appetitive LTM function; 5- Injection of *senior* animals with the SSRI fluoxetine reversed the effect of age on resting membrane potential of CGCs in a behavioural conditioning-independent manner while raising CGC electrical activity in behaviourally conditioned animals and restored appetitive LTM function.

### Relation to previous work on appetitive LTM conditioning in *Lymnaea stagnalis*

The current data are consistent with and corroborate several previous observations. First, by reproducing the results from an earlier study [6], the current data demonstrate that appetitive LTM impairment is a recurrent and reproducible phenomenon in aging *Lymnaea*. Moreover, the current results corroborate Kemenes et al.’s conclusion that CGC depolarization is a key factor in the expression of LTM in non-senescent animals [24]. Also, the observations that *senior* CGCs are electrically less excitable, with substantially lower action potential activity as compared to their junior counterparts and that this reduced electrical activity can be reversed by treatment with the lipophilic anti-oxidant α-tocopherol duplicates results we obtained on a different type of identified neuron in *Lymnaea*[3,6]. Thus, the current data support our idea that declining electrical excitability due to oxidative stress in the neuronal lipid-domain is a defining feature of the neurophysiology of aging in these snails. Lastly, our finding that fluoxetine treatment reversed the effect of age on CGC electrophysiology and appetitive LTM function complements Patel et al.’s observations that CGC’s (pre)synaptic functions change with age [26,27].

### Neurophysiological mechanisms of age-associated appetitive LTM impairment

The data presented here provide compelling evidence that CGC synaptic terminals are a key locus of the weakening appetitive LTM performance of aging *Lymnaea*. Moreover, our results suggest that this phenomenon has multiple dimensions. For instance, it is clear that chemosensory behavioural conditioning of old animals does not induce the delayed-onset, persistent depolarization of the CGCs critical for the presynaptic facilitation underlying expression of appetitive LTM [24]. Although the precise neural and signal transduction pathways involved in this process are still under investigation, it appears that behavioural-conditioning invoked cAMP/PKA-dependent recruitment of a persistent TTX-insensitive Na^+^ current (*I*_Na(p)_) is the first and critical step in a multi-tiered process leading to the facilitation of cerebral-buccal interneurons relaying peripheral chemosensory information to the buccal feeding circuit [24,28-30]. It is evident that this mechanism fails in old animals. Although we currently do not know for sure why this happens, some scenarios appear more likely than others. For instance, there is little evidence for peripheral chemosensory deficits in old animals this study, [6,31]. Therefore, although we can at this time not entirely exclude a role for primary chemosensory insufficiency as an explanation for age-associated appetitive LTM impairment it seems highly unlikely. Likewise, in contrast to other investigators working with this model system [23], we never found any evidence for major motor deficiencies in the feeding behaviour of old animals [6]. Nonetheless, the notion that primary chemosensory and motor functions of the buccal feeding complex appear to be essentially spared over the course of aging does not exclude the possibility that old animals integrate chemosensory information in a different fashion and relay this information in a different way to the feeding network. In fact, the literature does provide some precedents for that idea [6,27,31]. Moreover, such a scenario also appears consistent with the dramatically diminished excitability of aged CGCs, although it is not immediately evident how to reconcile this hypothesis with the observation that α-tocopherol did not restore LTM function in *senior* animals despite reversing the effects of age on CGC excitability.

### Mechanisms of CGC membrane depolarization

We show that both dietary supplementation of α-tocopherol and injection with fluoxetine caused depolarization of CGCs in aged animals. Because of their very different biological activities, this effect likely involves different mechanisms. As a chain-breaking lipophilic antioxidant α-tocopherol has the ability to intercept lipid peroxidation cascades and prevent further oxidation of the plasma membrane [32,33]. Our previous work has implicated lipid-peroxidation as a starting point for as yet incompletely understood mechanisms involved in the control of neuronal excitability [3,19]. For instance, various species of ion channels involved in the regulation of neuronal excitability are subject to redox-modulation [34-39]. Alpha-tocopherol could thus directly affect intrinsic excitability of neurons by interfering with ion channel oxidation status. Alternatively, because of its amphiphilic nature and structural asymmetry, α-tocopherol has the capacity to effect plasma membrane architectural changes, particularly changes in membrane curvature [40]. Membrane curvature is a variable in many membrane-associated processes including ion channel gating that could be a factor in the electrophysiological effects of α-tocopherol described here [41,42]. In addition, although we have not yet fully identified the signalling pathways involved, our own work indicates that α-tocopherol can intervene in neuronal lipid signalling processes that are apparently associated with reduced excitability of aged neurons [3,19]. Thus, α-tocopherol likely operates primarily on plasmamembrane- or lipid signalling-associated determinants of intrinsic neuronal excitability [3,19]. Fluoxetine on the other hand, may induce CGC membrane depolarization by augmenting autoregulatory feedback mechanisms. The cells express one or more of the G_i_-coupled serotonin receptor subtypes that in the case of serotonin-dependent presynaptic facilitation of sensory pathways in the *Aplysia californica* gill-withdrawal reflex model have been implicated in the closure of S-like K^+^ channels [43-47]. Parenthetically, there is some evidence that these channels, members of the 4 transmembrane, 2 pore-region (4TM2P) family of background K^+^ channels are directly inhibited by fluoxetine and its metabolites [47,48]. It should be noted however that recent studies suggest that fluoxetine’s spectrum of pharmacological activities in the *Lymnaea* nervous system extends beyond the compound’s “classical SSRI activity. [26,49]. Therefore, further studies are required to examine the mechanisms through which fluoxetine ameliorates the effects of age on neural- and behavioural plasticity in this model system.

### Why does dietary supplementation of α-tocopherol fail to alleviate age-associated LTM impairment while it did reverse the effects of age on neuronal excitability?

One of the remarkable aspects of our results is the differential ability of α-tocopherol and fluoxetine to reverse both electrophysiological and behavioural effects of age. That is, the results show that dietary supplementation of α-tocopherol successfully reversed the effect of age on CGC resting membrane potential and electrical activity but failed to alleviate LTM impairment in old animals whereas a single injection of fluoxetine reversed both electrophysiological and behavioural effects of age. Alpha-tocopherol’s inability to restore LTM function in *senior* animals is particularly puzzling since experimental depolarization of CGCs in semi-intact preparations from, presumably, non-senescent animals was shown to be sufficient to replicate the effect of behavioural conditioning on feeding network responses to peripheral chemosensory stimuli [24]. Thus, we are left with the conclusion that in *Lymnaea* neurons, treatment with α-tocopherol may reverse low-excitability conditions associated with old age but does not bring about facilitation of neurotransmitter release while fluoxetine is, at least in the case of the CGCs, apparently capable of both. We currently have no definitive explanation for this dissimilarity. It is possible that fluoxetine, being a SSRI, augments CGC synaptic efficacy through enhancing postsynaptic serotonergic mechanisms. In addition, as discussed above, it is possible that fluoxetine induce CGC membrane depolarization by augmenting autoregulatory feedback mechanisms. That is, there is some evidence suggesting that serotonin acts as an excitatory autotransmitter in CGCs through mechanisms analogous to the serotonin-dependent presynaptic facilitation of sensory pathways previously described in the *Aplysia californica* gill-withdrawal reflex model [43-47]. Also, the literature hints at an alternative presynaptic mechanism. That is, Shomrat et al. [50] demonstrated that serotonin facilitates induction of activity-dependent long-term potentiation in *Octopus vulgaris*. In addition, Hart et al. [51] recently implicated synapsin, a protein critical in the regulation of vesicle recruitment and pre-synaptic neurotransmitter release, in 5-HT-dependent long-term presynaptic facilitation in sensorimotor synapses of the gastropod *Aplysia californica* through the modulation of vesicle release mechanisms. This finding is particularly interesting in the context of the present study, since Patel et al. [26] reported that fluoxetine reverses age-associated effects of neurotransmitter release by CGCs. Although it is not yet clear through which mechanism(s) synapsin promotes secretory vesicle release probability, these two studies suggest that, in contrast to α-tocopherol, fluoxetine promotes presynaptic facilitation of CGCs and thereby improves LTM function in our aged animals through a depolarization-independent presynaptic mechanism. One of the intriguing implications of this idea is that synapsins may be a target for treatment of age-associated cognitive impairment. Further studies are needed to answer this question and to examine whether this principle can be generalized to different types of chemical synapses.

An alternative explanation for α-tocopherol’s failure to reproduce the presynaptic facilitation of CGC synaptic terminals required for the expression of appetitive LTM in old animals worthwhile considering centers on the possibility of an age-associated breakdown of background Ca^2+^ signalling mechanisms. Modulation of neurotransmitter release through variation of presynaptic resting membrane potential proceeds through small changes in intracellular free Ca^2+^ concentrations arising from subthreshold activation of voltage-gated Ca^2+^ channels [52]. There is evidence suggesting that a similar mechanism may contribute to presynaptic facilitation in *Lymnaea* CGCs [24]. Changes in intracellular Ca^2+^ homeostasis are quite a common symptom of neuronal aging [53]. It is therefore possible that the differential effectiveness of α-tocopherol and fluoxetine in restoration of appetitive LTM in old animals could be a reflection of the fact that the former depends mostly on background Ca^2+^ signalling mechanisms whereas the latter, as discussed above, may not.

Taken together, whereas the current data establish the CGC as a major neurophysiologic correlate of age-associated LTM dysfunction in *Lymnaea* and does reiterate the significance of lipid (per)oxidation in neuronal excitability decline with age, it is evident that further investigations are needed to resolve exact causal relations between one and the other.

## Conclusions

Although we have not yet identified the precise molecular mechanisms, our data leaves little doubt that the CGCs are a locus of age-associated LTM-impairment in *Lymnaea.* As such, our study identifies failure of experience-induced, depolarization-dependent presynaptic facilitation of the CGCs as a very probable neurobiological correlate of this memory defect. Moreover, the current results further underpin the conclusion from our previous work that lipid-peroxidation, likely through activation of phospholipase A_2_ (PLA_2_), is a pivotal factor in the gradual decline in neural- and behavioral plasticity associated with the normal aging process [3,19]. It is important to recognize that we do not stand alone in this conclusion. The significance of lipid-peroxidation in aging in phylogenetically quite diverse model systems has been acknowledged for a long time [54-57]. Moreover, recent reports explicitly associate PLA_2_-activity with age-associated cognitive deficits and neurodegenerative diseases [58]. Thus, we believe that the phenomena described here reflect the actions of universal, evolutionary conserved mechanism of neuronal aging originating in lipid peroxidation. It is evident from the work presented here that *Lymnaea’s* feeding behavior and underlying neural system provides a superbly traceable model to dissect the neurobiological substrate of learning and memory impairment associated with normal aging.

## Methods

### Animal populations

Animals were bred and raised under constant conditions as previously described [3,6,19,59]. Snails were raised in house under strictly controlled ambient conditions (light:dark 12:12, ambient temperature 18-19°C, pH 7.8-7.9). Water used in the facility was sourced from a reverse osmosis system and reconditioned to a conductivity of ~450 Ω.cm through the addition of Instant Ocean salts at 1 g/US Gallon (i.e., artificial pond water; Aquarium Systems USA). Calcium concentration was kept at saturation level (~60 mg/L as CaCO_3_) through the addition of calcium carbonate (light powder; EMD analytics, Gibbstown, New Jersey) to the tanks. In addition, animals had continuous access to sterilized cuttlefish (*Sepia officinalis*) bone (2–3 per tank). Unless stated differently, animals in this study were fed a routine diet consisting of Romaine lettuce and Aquamax-carniverous Grower 600 trout pellets *ad libitum* (Purina Mills LLC, St. Louis, Missouri).

Survival characteristics of the populations were continuously monitored and evaluated using previously established methods based on the Weibull failure model (Table1) [60,61]. Experimental animals were taken at random from different age-synchronized healthy, populations with survival percentages at the time of sampling ranging from >99% to <25% survival (Table1). For the purpose of this study, old (“*senior*”) animals (18–23 months of age) are defined as animals falling in the 25% or less survival range, while sexually mature young adult (“*junior*”) animals (5–7 months of age) fall into the 95% or more survival range. Survival characteristics as defined by Weibull model parameters (see Table1) of all populations used in this study closely aligned with those used in earlier studies [3,6,19]. 

**Table 1:** **Parameter estimates of two-parameter Weibull failure model fitted to the survival characteristics of *Lymnaea stagnalis *populations used in the present study**

**Population**	***a*(error)**	***c*(error)**	***s*· 10**^**-2**^**(error)**	**% survival at sampling**	**age range (month)**
Junior 1	490 (9.9)	3.7 (0.25)	−0.26 (1.37 · 10^-2^)	>95%	5-7
Senior 1	440 (8.3)	4.3 (0.29)	−0.34 (1.19 · 10^-2^)	<25%	18-20
Senior 2	550 (9.6)	4.0 (0.29)	−0.25 (1.56 · 10^-2^)	<25%	21-22
Senior 3	550 (7.4)	4.5 (0.29)	−0.28 (1.91 · 10^-2^)	<25%	21-22
Senior 4	570 (9.6)	3.8 (0.28)	−0.23 (1.66 · 10-^2^)	<25%	22-23

Note: “*a*” and “*c*” are parameters of the two-parameter Weibull model (“*a*” is median age; “*c*” is the shape parameter; “*s*” is slope of tangent of survival curve at median age).

The use and care of animals conformed to the University of Calgary Animal Care and Use Policy which adheres to the guidelines, policies and standards of the Canadian Council on Animal Care (CCAC), the Canadian Association of Laboratory Animal Medicine (CALAM), standards of Veterinary Care, and the Alberta Veterinary Association (AVMA) professional codes and standards.

### Training and testing procedures

#### Preparation

Behavioural conditioning was done on 3 to 4 sets of animals each constituted of equal numbers of individuals in an experimental groups (the number of replicate training sessions depended on the sample size required) using a non-aversive appetitive classical conditioning protocol modified from Hermann et al. [6]. Snails from each of the two age cohorts were sampled at random. For identification purposes a small indelible label was glued to the shell of each snail or marked with indelible marker. Except during testing and training periods, the snails were housed in a common 100-L “home” tank filled with artificial pond water. Food was withheld starting 48 hrs prior to the first pre-training test and for the remainder of the training and post-training testing.

#### Testing procedures

Animals from different age and experimental groups were individually tested in random order. On day 1, prior to behavioural conditioning each snail was individually tested for their natural response to the administration of pond water, the disturbance stimulus (DS), as well as the conditional stimulus (CS) n-amyl acetate (4 ppm final concentration, Figure1A, “Pre-training test”; see also [6]). Tests were performed using 100-ml translucent polystyrene beakers (4.5 cm diameter), filled with 80 mL of water taken from the snail’s home tank. After transfer into the beakers, the snails were allowed to acclimatize for 15 min before testing commenced. Testing involved counting the number of rasps over two consecutive periods of 2 min, the first period starting with gentle administration of the DS (10 ml artificial pond water), the second period starting with the administration of the CS (10 ml n-amyl acetate solution). To facilitate observation, the test beakers were elevated by translucent plastic stands and were surrounded by mirrors to ensure continuous view of the buccal mass and radula of the snails during testing. A test response was calculated by taking the difference between the number of rasps counted during the second period minus the number counted during the first period (i.e., ΔRasp = rasps after CS – rasps after DS). To correct for both differences in background rasping activity and potential application artefacts, the pre-training tests were performed in duplicate with >1 hr interval, and behavioural responses were calculated as the average of both ΔRasp (i.e., ΔRasp_pre-test_ = average ΔRasp_pre-test_1 and ΔRasp_pre-test_2). To determine in some experiments non-associative rasping behaviour, we also counted for a period of 2 minutes the number of rasps before DS application. Thus the disturbance (DS) response is: ΔRasp_DS_ = rasps after DS - rasps before DS. After completion of a test, snails were gently rinsed and returned to their home tanks. A single post-training test was performed on day 3 (i.e., 24 hrs after training) following identical procedures as described for the pre-testing above (Figure1A).

#### Training procedure

Snails were trained in a single day, multi-trial forward-delay conditioning format (Figure1A; modified from [6]). Sucrose (final concentration of 0.4% wt/vol) served as the unconditioned stimulus (UCS) and n-amyl acetate (4 ppm final concentration) as the conditioned stimulus (CS). To control for potential behavioural effects of fluid addition, a disturbance control in which the UCS was paired with the DS (i.e., pond water) was implemented. Snails were randomly assigned to either the CS–UCS (“conditioned”) or the CS–DS (unconditioned “control”) group and trained “en masse”. Training was performed in 1-L polypropylene beakers containing 480 ml clean artificial pond water. After transfer into the training beakers, the snails were allowed to acclimatize for 60 min. Both “control” and “conditioned” groups received 120 mL of the CS solution, followed 15 s later by 120 ml of the UCS (“conditioned” group) or 120 ml of the DS (“control” group). After 2 min, the beakers containing the snails were drained and gently rinsed with clean pond water and the snails were readied for their next training trial by re-placing them in the 1-L polypropylene beakers holding 480 ml clean artificial pond water. After 11 min and 45 sec the training procedure was repeated (Figure1A). Snails received a total of 5 training procedures on a single day before being returned to their “home” tank. During training and testing, snails were at all times fully submerged. Care was taken to ensure that pre-testing, training and post-testing commenced at the same time of day for each group and training and testing always occurred in the same location. “Conditioned” and “control” snails were always tested and trained concurrently.

### Anti-oxidant supplementation

Snails 16–17 month of age were sampled at random from their parent populations, labelled, housed in a common 100-L “home” tank and food deprived for 48 hrs before being submitted to behavioural assessments. After the post-test, animals were transferred to 15-L polypropylene containers filled with artificial pond water and subdivided in 12 equal-sized compartments created with materials allowing free flow of water that contained one snail each. This arrangement allowed for individual feeding of snails, yet maintained otherwise uniform environmental conditions for the different test groups. Snails were randomly split into two test groups. Both groups were fed lettuce *ad libitum*. In addition, one group received agar pellets (3% w/v) containing α-tocopherol dissolved in EtOH (0.13% v/v; 0.08 mg α-tocopherol/pellet) plus Nutrafin Basix fish flakes (0.04 mg/pellet), whereas the other group received agar pellets containing Nutrafin Basix fish flakes only (Note, EtOH is evaporated during the preparation of the agar pellets). Each snail received a single pellet every 48 hrs. After a period of 2 months, the snails were transferred to the common 100-L “home” tank, food deprived for 48 hrs and subsequently trained and tested to re-assess LTM performance as described before, with each snail staying within their previously assigned groups (i.e., either CS + UCS or CS + DS).

### Fluoxetine treatment

Fifty microliters of fluoxetine dissolved in ultrapure H_2_O was injected into the animal creating an approximate final concentration of 100nM. Another set of animals received an injection of 50 μl ultrapure H_2_O only (vehicle control). Injection was done 2Â½ hrs prior to the start of the first training session. All solutions were injected through the foot directly into the hemocoel with a microliter syringe and a 30 G needle. Animals were behaving normally within a few minutes after injection.

### Unconditioned sucrose induced feeding behaviour

To determine whether α-tocopherol caused a chemosensory and/or motor deficiency, naïve (unconditioned) animals were tested for their feeding response upon sucrose application. Tests were performed using 100-ml translucent polystyrene beakers (4.5 cm diameter), filled with 80 mL of water taken from the snail’s home tank. After transfer into the beakers, the snails were allowed to acclimatize for 15 min before testing commenced. Testing involved counting the number of rasps over three consecutive periods of 2 min, the second period starting with gentle administration of artificial pond water (10 ml) a disturbance stimulus (DS), the third period starting with the administration of sucrose solution (0.4% w/v final concentration). The disturbance response was calculated by taking the difference between the number of rasps counted during the second period minus the number counted during the first period (i.e., ΔRasp disturbance = rasps after DS – rasps after pond water). The sucrose response was calculated by taking the difference between the number of rasps counted during the third period minus the number counted during the second period (i.e., ΔRasp surcose = rasps after sucrose – rasps after DS).

### Electrophysiology

#### Intracellular recordings

Both conditioned and control snails were dissected and subjected to electrophysiological evaluation within 48 hrs of final behavioural assessment (post-training test). To this end, snails were de-shelled using curved forceps and subsequently anaesthetized in a 25% Listerine solution. All dissections and experiments were carried out in a hydroxyethylpiperazine ethanesulphonic acid (HEPES) -buffered saline (HBS) composed of (in mM) 51.3 NaCl, 1.7 KCl, 4.1 CaCl_2_, 1,5 MgCl_2_ and 10 HEPES (pH 7.9). CNSs were dissected as described previously [60]. Dissected CNSs were pinned down in an elastomer covered recording chamber filled with HBS. To provide access to the Cerebral Giant Cells (CGCs), the outer layer of connective tissue surrounding the cerebral ganglia were removed with fine forceps without the use of proteolytic enzymes [62,63]. Intracellular recordings of the CGCs were done by means of microelectrode recording techniques with the use of either Axoclamp 2A or Axoclamp 2B, (Axon Instruments, Burlingame, CA) operated in discontinuous current clamp mode at sample rates ranging from 2–4 kHz depending on electrode settling characteristics. Amplifier output was filtered at 1 kHz and digitized at 5 kHz using a Digidata 1322A AD/DA converter (Axon Instruments, Burlingame, CA). Data acquisition was carried out with Axoscope sampling software (version 9.0, Axon Instruments) and subsequently analyzed using Clampfit software (version 9.0 Axon Instruments). Borisilicate glass electrodes (TW150F, World Precision Instruments, Sarasota, FL) were filled with a solution of 0.5 M potassium acetate (CH_3_COOK) and 0.01 M potassium chloride (KCl). DC resistance of the electrodes ranged between 10–25 MΩ.

#### Extracellular recordings

Extracellular recordings of lip sensory neurons were made from the superior lip nerve by means of a suction electrode in a semi-intact preparation as described by Straub et al. [64]. Briefly, after snails were anaesthetized with 25%Listerine solution, the central nervous system was exposed by means of a small dorsal incision in the midline of the head region. The head of the snail containing the lips, tentacles and buccal mass, plus the CNS was carefully dissected from the rest of the body. Subsequently the right superior lip nerve was cut as close to the CNS as possible. The remaining peripheral nerves were cut close to their muscle innervation zone and the CNS was discarded. The preparation was pinned down in a Sylgard coated dish, and the superior lip nerve was drawn into a glass microelectrode with a diameter just large enough to accommodate the nerve. A perfusion system was used to continuous apply saline or n-amyl acetate (4 ppm) with the outflow placed close to the lip/mouth region. Extracellular signals were amplified using an in-house build extracellular amplifier, bandpass filtered at 10–1000 Hz and digitized at 5 kHz sampling rate using a Digidata 1322A AD/DA converter (Axon Instruments, Union City, CA). Recordings were analyzed offline using template-matching waveform recognition software (Spike2, version 4.02a, Cambridge, England). Template selection criteria were set to ignore waveforms that occurred less frequently than 1 in 50 events. From these data overall peristimulus histograms were generated with a bin size of 30 seconds. The data was normalized to the average activity of the period prior to amyl acetate application.

### Statistical analysis

Behavioural data was analyzed using repeated measures ANOVA. Spontaneous activity of the CGCs was analyzed by means of a factorial ANOVA. Explicit hypotheses were tested using linear contrast techniques unless indicated differently in the text. Compliance with parametric assumptions was confirmed for each data set submitted to ANOVA using both graphical (probability plots applied to raw data and residuals) and analytical techniques (Kolmogorov-Smirnov one-sample test for normality, F-max test). Nonparametric tests were used to analyze CGC resting membrane potential (Mann–Whitney *U* test or Kruskal-Wallis). Throughout the text, average and data dispersion are expressed as arithmetic means and standard error of the mean (SEM).

## Abbreviations

LTM: Long-term memory; DS: Disturbance stimulus; CS: Conditioned stimulus; UCS: Unconditioned stimulus; PLA2: Phospholipase A2; HBS: HEPES buffered saline; CGC: Cerebral giant cell; SSRI: Selective serotonin reuptake inhibitor.

## Competing interests

The authors declare that they have no competing interests.

## Authors’ contributions

Conceived research program WCW. Conceived and designed the experiments: SNW, TER, PMH, WCW. Performed the experiments: SNW, TER, PMH. Analyzed the data SNW, TER, PMH, WCW. Wrote the paper: SNW, TER, PMH, WCW. All authors read and approved the final manuscript.
